# Oral Contraceptive Use Associated With Lower Rates of Knee Instability After Anterior Cruciate Ligament (ACL) Reconstruction

**DOI:** 10.7759/cureus.110003

**Published:** 2026-05-31

**Authors:** Ronak J Mahatme, Shawn A Moore, Anish Gangavaram, Nishanth Muthusamy, Esha Reddy, Samuel K Gerak, Brian M Grawe

**Affiliations:** 1 Department of Orthopaedics, University of Cincinnati College of Medicine, Cincinnati, USA; 2 Division of Sports Medicine, Department of Orthopaedic Surgery, University of Cincinnati College of Medicine, Cincinnati, USA

**Keywords:** anterior cruciate ligament reconstruction, estrogen, knee instability, opioid use, oral contraceptives

## Abstract

Background

Sex hormones influence connective tissue biology and pain processing, but the relationship between oral contraceptive pill (OCP) use and postoperative outcomes after anterior cruciate ligament reconstruction (ACLR) remains unclear. The purpose of this study was to evaluate whether perioperative OCP use is associated with postoperative knee instability and other clinical outcomes after ACLR.

Methods

Using a multicenter database, TriNetX, females aged 14-40 years undergoing arthroscopic ACLR were grouped as OCP users (prescriptions within six months before and within two years after surgery) or non-users (no OCP prescriptions from one year before to two years after surgery). Propensity score matching (1:1) based on demographics and comorbidities yielded 815 patients per cohort. Primary outcomes were two-year reoperation, chronic knee instability diagnosis, and chronic knee pain diagnosis. Secondary outcomes included 30-day surgical site infection (SSI), 90-day deep or organ infection, six-month arthrofibrosis/manipulation under anesthesia (MUA), and postoperative opioid use (early, 1-30 days; prolonged, 30-90 days; chronic, 90-180 days). Risk ratios (RRs) with 95% confidence intervals (CIs) were calculated.

Results

OCP use was associated with significantly lower rates of two-year chronic knee instability (RR, 0.316; 95% CI, 0.166-0.600; p < 0.001) and early postoperative opioid use (RR, 0.363; 95% CI, 0.281-0.468; p < 0.001). Rates were similar between groups for six-month arthrofibrosis/MUA (RR, 0.750; 95% CI, 0.357-1.575; p = 0.446), two-year reoperation (RR, 1.057; 95% CI, 0.735-1.519; p = 0.766), two-year chronic knee pain (RR, 0.976; 95% CI, 0.877-1.086; p = 0.654), 30-day SSI (rare), and 90-day deep or organ infection (none; p = 1.000).

Conclusion

In a large propensity-matched cohort of female patients undergoing ACLR, perioperative OCP use was associated with markedly lower rates of two-year knee instability and reduced early opioid use, whereas other complications and reoperation rates did not differ, highlighting hormonal status as a potentially modifiable factor in postoperative recovery.

## Introduction

Although oral contraceptive pill (OCP) use has declined in recent years, it remains one of the most common forms of contraception in the United States [1,2]. Many OCP formulations contain estrogen in combination with progestins, preventing pregnancy primarily by suppressing ovulation [3]. Beyond reproductive effects, estrogen exerts wide-ranging physiologic influence across cardiovascular, gastrointestinal, and musculoskeletal systems [4,5] and plays a complex role in wound healing and local inflammatory processes [6,7].

Anterior cruciate ligament (ACL) injuries are among the most frequent ligamentous injuries in young athletes, with women experiencing rates up to four to six times higher than men in comparable sports [8]. This disparity has spurred growing interest in the role of sex hormones in both injury risk and recovery. Most existing research on OCPs in orthopedics has focused on injury prevention, particularly the relationship between menstrual cycle hormonal fluctuations and ACL rupture risk [9]. However, less is known about how OCP use influences postoperative outcomes after ACL reconstruction (ACLR), especially regarding graft healing and knee stability.

Estrogen’s involvement in inflammatory signaling, angiogenesis, and collagen remodeling suggests that hormonal status may influence graft incorporation and ligament remodeling after ACLR [10,11]. Early evidence from human and animal studies indicates that estrogen can affect tendon collagen synthesis, fibril organization, and biomechanical strength, potentially altering postoperative stability [12,13]. Nevertheless, existing studies are often limited by small sample sizes, heterogeneous surgical cohorts, and incomplete characterization of OCP timing and duration.

Given the importance of achieving long-term knee stability and pain-free function after ACLR, understanding modifiable perioperative factors such as hormonal environment could inform strategies to optimize outcomes. The present study investigates whether combined estrogen-progestin or estrogen-only OCP use, both before and after surgery, is associated with postoperative outcomes following ACLR. We specifically evaluated two-year knee instability, reoperation, and chronic knee pain as primary outcomes, with secondary analyses including postoperative infection, arthrofibrosis, and opioid use. We hypothesized that OCP use would be associated with differences in postoperative knee instability and other adverse outcomes compared with non-use.

## Materials and methods

Data source

The data used in this retrospective cohort study were collected on August 6, 2025, from the TriNetX Research Network [14], which provides access to electronic medical records (diagnoses, procedures, medications, laboratory values, and genomic information) from more than 150 million patients across 102 healthcare organizations. TriNetX, LLC (Cambridge, MA) [14] is compliant with HIPAA (Health Insurance Portability and Accountability Act) and applicable data privacy regulations, and all data accessed through the platform is de-identified in accordance with federal standards. As this study used only de-identified patient records and did not involve the collection, use, or transmittal of individually identifiable data, it was exempt from the Institutional Review Board approval.

Cohort selection and inclusion criteria

Two patient cohorts were identified. The control group (OCP-) included patients aged 14-40 years at the time of arthroscopic ACLR who had no record of oral contraceptive use within one year before or two years after surgery. The experimental group (OCP+) included patients aged 14-40 years who were prescribed oral contraceptives both within six months before surgery and within two years after surgery. To specifically evaluate the effects of exogenous estrogen, only patients prescribed combined estrogen-progestin or estrogen-only formulations were included in the OCP+ group.

Exclusion criteria and matching variables

Patients were excluded if, within six months before ACLR, they had a diagnosis of pregnancy, childbirth, polytrauma, or had undergone surgical procedures on the femur or knee joint. Additional exclusions included osteoporosis with current pathological fracture, malignant neoplasms of bone or articular cartilage, prior knee arthroscopy, systemic connective tissue disorders, amenorrhea, oligomenorrhea, or polycystic ovarian syndrome. Propensity score matching (1:1) was performed using the TriNetX algorithm, accounting for the following variables: age at index surgery, sex, race, body mass index (BMI > 30), tobacco use, osteoporosis without current pathological fracture, vitamin D deficiency, chronic kidney disease, and heart failure (Table 1). This resulted in matched cohorts of 815 patients each. The platform integrates the nearest-neighbor matching with a tolerance level of 0.01 and ensures that the difference between propensity scores is P ≤ 0.1. Post-matching covariate balance was assessed using standardized mean differences (SMD), with SMD < 0.1 indicating appropriate balance between cohorts, which is consistent with TriNetX validation standards [14].

**Table 1:** Patient demographics and propensity score matching of OCP+ and OCP- patients undergoing arthroscopic ACLR OCP: Oral contraceptive pills; BMI: Body mass index; CKD: Chronic kidney disease.

	Unmatched Cohort			Matched Cohort		
Characteristics	OCP+ (% of Cohort)	OCP- (% of Cohort)	P-Value	OCP+ (% of Cohort)	OCP- (% of Cohort)	P-Value
Total	819 (100%)	27,786 (100%)		815 (100%)	815 (100%)	
Age at index	24.7 ± 6.9 (100%)	23.4 ± 8.0 (100%)	<0.001	24.7 ± 6.9 (100%)	24.6 ± 6.9 (100%)	0.903
Race						
White	673 (82.5%)	18,295 (68.1%)	<0.001	672 (82.5%)	675 (82.8%)	0.844
Black or African American	46 (5.6%)	3,011 (11.2%)	<0.001	46 (5.6%)	44 (5.4%)	0.828
Asian	23 (2.8%)	1,224 (4.6%)	0.019	23 (2.8%)	23 (2.8%)	1
American Indian or Alaska Native	10 (1.2%)	247 (0.9%)	0.368	10 (1.2%)	10 (1.2%)	1
Native Hawaiian or other Pacific Islander	10 (1.2%)	374 (1.4%)	0.689	10 (1.2%)	10 (1.2%)	1
Other race	35 (4.3%)	1,346 (5.0%)	0.353	35 (4.3%)	35 (4.3%)	1
Female	816 (100%)	26,876 (100%)	--	815 (100%)	815 (100%)	--
Male	0 (0%)	0 (0%)	--	0 (0%)	0 (0%)	--
Diagnosis						
Body mass index (BMI) 30-39, adult	26 (3.2%)	542 (2.0%)	0.020	26 (3.2%)	28 (3.4%)	0.782
Body mass index (BMI) 40 or greater, adult	10 (1.2%)	339 (1.3%)	0.928	10 (1.2%)	12 (1.5%)	0.668
Osteoporosis without a current pathological fracture	0 (0%)	13 (0.0%)	0.530	0 (0%)	0 (0%)	--
Vitamin D deficiency	30 (3.7%)	626 (2.3%)	0.013	30 (3.7%)	27 (3.3%)	0.686
Tobacco use	10 (1.2%)	182 (0.7%)	0.063	10 (1.2%)	10 (1.2%)	1
Diabetes mellitus	10 (1.2%)	181 (0.7%)	0.061	10 (1.2%)	10 (1.2%)	1
Chronic kidney disease (CKD)	10 (1.2%)	41 (0.2%)	<0.001	10 (1.2%)	10 (1.2%)	1
Heart failure	10 (1.2%)	18 (0.1%)	<0.001	10 (1.2%)	0 (0%)	--

Outcome measures 

Primary outcomes were two-year rates of reoperation, chronic knee instability diagnosis, and chronic knee pain diagnosis. Secondary outcomes included 30-day surgical site infection (SSI), 90-day deep/organ infection, six-month arthrofibrosis or manipulation under anesthesia (MUA), and postoperative opioid use.

All outcomes of interest were identified using current procedural terminology (CPT), a standardized procedural coding system maintained by the American Medical Association; International Classification of Diseases, 10th Revision, Clinical Modification (ICD-10-CM), a standardized diagnostic coding system maintained by the Centers for Disease Control and Prevention; and RxNorm codes, a standardized medication nomenclature developed by the National Library of Medicine [15-17]. These coding systems have been widely used in prior orthopedic database research; however, formal validation for specific postoperative ACLR outcomes within TriNetX remains limited. All codes used for postoperative outcomes are provided in the Appendix.

Risk ratios (RRs), absolute risk differences, and 95% confidence intervals (CIs) were computed, and complication rates were analyzed using the TriNetX system [14]. Categorical variables were assessed using the chi-squared test, whereas continuous variables were evaluated using independent t-tests. Statistical significance was set at P < 0.05. Because multiple outcomes were evaluated, the findings should be interpreted as exploratory and hypothesis-generating.

## Results

Cohort identification and matching

Before propensity score matching, the OCP+ cohort (patients aged 14-40 years undergoing arthroscopic ACLR with oral contraceptive use within six months before and two years after surgery) included 819 patients. The OCP- cohort (patients aged 14-40 years with no oral contraceptive use within one year before or two years after surgery) included 27,786 patients. After 1:1 propensity score matching, 815 patients remained in each group for analysis.

30-day, 90-day, and two-year outcomes

**Figure 1:**
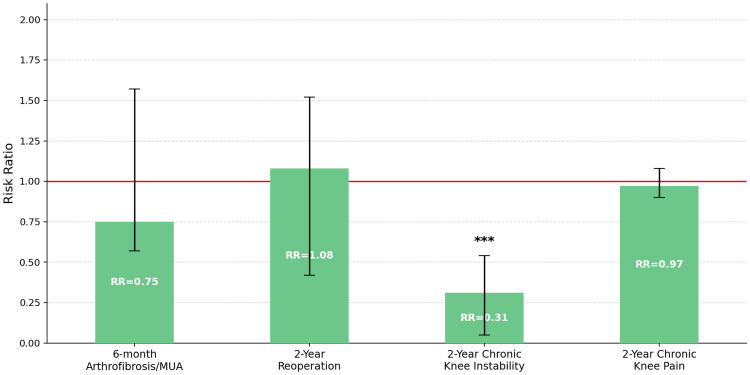
Risk ratios comparing six-month and two-year outcomes between OCP+ and OCP- cohorts undergoing arthroscopic ACLR * P < 0.05; ** P < 0.01; *** P < 0.001. OCP: Oral contraceptive pill; ACLR: Anterior cruciate ligament reconstruction; MUA: Manipulation under anesthesia.

No 30-day surgical site infections occurred in the OCP+ group, and ≤10 events occurred in the OCP- group, limiting statistical analysis and interpretation of infection risk. No 90-day deep or organ infections were observed in either group (p = 1.000). Rates of six-month arthrofibrosis or MUA (RR, 0.750; 95% CI, 0.357-1.575; p = 0.446), two-year reoperation (RR, 1.057; 95% CI, 0.735-1.519; p = 0.766), and two-year chronic knee pain (RR, 0.976; 95% CI, 0.877-1.086; p = 0.654) were similar between groups. However, two-year chronic knee instability was significantly lower in the OCP+ group (1.5% vs 4.7%; absolute risk reduction, 3.2%; RR, 0.316; 95% CI, 0.166-0.600; p < 0.001) (Figure 1 and Table 2).

**Table 2:** 30-day, 90-day, six-month, and two-year outcomes between OCP+ and OCP- cohorts undergoing arthroscopic ACLR * Event counts < 11 are rounded per TriNetX reporting policies to prevent patient re-identification. SSI: Surgical site infection; OCP: Oral contraceptive pill; ACLR: Anterior cruciate ligament reconstruction; MUA: Manipulation under anesthesia.

Variables	OCP+ Events (Risk %)	OCP- Events (Risk %)	Total Patients Per Cohort	Risk Ratio (95% CI)	P-Value
30-day SSI	0 (0.0)	≤10* (1.2)	815	-	-
90-day deep/organ infection	0 (0.0)	0 (0.0)	815	-	1.000
Six-month arthrofibrosis/MUA	12 (1.5)	16 (2.0)	815	0.750 (0.357-1.575)	0.446
Two-year reoperation	56 (6.9)	53 (6.5)	815	1.057 (0.735-1.519)	0.766
Two-year chronic knee instability	12 (1.5)	38 (4.7)	815	0.316 (0.166-0.600)	<0.001
Two-year chronic knee pain	363 (44.5)	372 (45.6)	815	0.976 (0.877-1.086)	0.654

Early, prolonged, and chronic postoperative opioid use 

**Figure 2:**
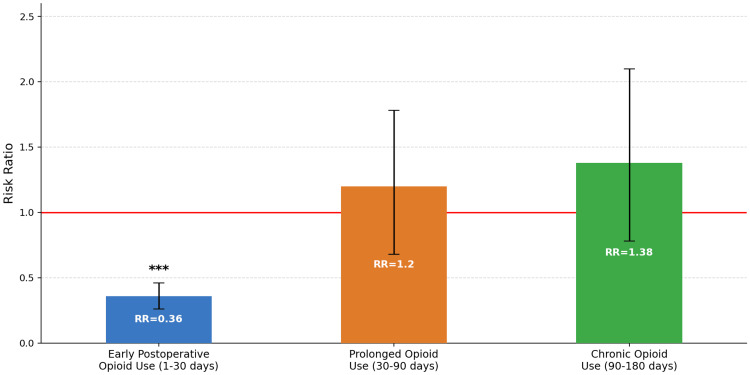
Risk ratios comparing opioid use between OCP+ and OCP- cohorts following arthroscopic ACLR * P < 0.05; ** P < 0.01; *** P < 0.001. OCP: Oral contraceptive pill; ACLR: Anterior cruciate ligament reconstruction.

Early postoperative opioid use (1-30 days) was significantly lower in the OCP+ cohort compared with the OCP- cohort (8.6% vs 23.7%; absolute risk reduction, 15.1%; RR, 0.363; 95% CI, 0.281-0.468; p < 0.001). No significant differences were observed between groups for prolonged postoperative opioid use (30-90 days) (RR, 1.209; 95% CI, 0.817-1.790; p = 0.341) or chronic postoperative opioid use (90-180 days) (RR, 1.400; 95% CI, 0.917-2.137; p = 0.117) (Figure 2 and Table 3).

**Table 3:** Early, prolonged, and chronic postoperative opioid use in OCP+ and OCP- patients undergoing arthroscopic ACLR OCP: Oral contraceptive pill; ACLR: Anterior cruciate ligament reconstruction.

Variables	OCP+ Events (Risk %)	OCP- Events (Risk %)	Total Patients Per Cohort	Risk Ratio (95% CI)	P-Value
Early postoperative opioid use (1-30 days)	70 (8.6)	193 (23.7)	815	0.363 (0.281-0.468)	<0.001
Prolonged postoperative opioid use (30-90 days)	52 (6.4)	43 (5.3)	815	1.209 (0.817-1.790)	0.341
Chronic postoperative opioid use (90-180 days)	49 (6.0)	35 (4.3)	815	1.400 (0.917-2.137)	0.117

## Discussion

In this retrospective cohort study, patients on OCPs both before and after ACLR demonstrated significantly lower rates of chronic knee instability at two years and reduced early postoperative opioid use. These findings demonstrate an association between recorded perioperative OCP use and selected postoperative outcomes after ACLR. Although biologic mechanisms involving estrogen signaling may contribute to these observations, the present study cannot establish causation or directly evaluate graft healing biology.

Knee instability after ACLR is multifactorial, involving factors such as incomplete graft integration, residual laxity, or deficits in neuromuscular control [18]. Estrogen influences tendon and ligament biology through modulation of collagen synthesis, regulation of inflammatory pathways, and effects on connective tissue remodeling [11]. Prior research has focused largely on endogenous hormonal fluctuations and their role in injury risk, with several studies reporting a modest protective effect of OCP use against ACL injury [19,20]. Our results raise the possibility that exogenous estrogen exposure may be associated with postoperative recovery differences after ACLR; however, the biologic basis of this association remains speculative. One potential explanation involves relaxin, a hormone associated with increased ligamentous laxity. Fluctuations in relaxin seen during the menstrual cycle, pregnancy, or perimenopause have been linked to changes in ACL and other ligament biomechanics [12]. By stabilizing hormone levels, OCPs may suppress relaxin variability, potentially reducing hormonally mediated ligament laxity. This hypothetical mechanism may partially explain the association with the lower rates of chronic instability observed in OCP users [12].

We also found that early postoperative opioid use was significantly lower among OCP users. While causality cannot be established, this association supports the idea that hormonal status influences pain perception and analgesic requirements in the immediate postoperative period. Estrogen modulates pain processing both peripherally and centrally through effects on glial activation, nociceptor sensitivity, and opioid receptor expression [21]. Estradiol has been shown to enhance mu-opioid receptor function, increasing opioid sensitivity and reducing the drive for opioid intake. Preclinical work demonstrates that estradiol replacement in ovariectomized rats increases the analgesic potency of fentanyl and decreases voluntary opioid consumption, suggesting enhanced endogenous opioid signaling [22]. In humans, Craft’s review found that estrogen can exert either pain-amplifying or pain-reducing effects depending on the pain condition and underlying physiological systems [23]. Because estrogens modulate nervous, immune, skeletal, and cardiovascular systems, their influence on pain regulation is complex and context-dependent. Additionally, since OCPs alter hormonal exposure patterns, it is possible that hormonal differences contributed to the observed association with postoperative opioid use. However, the current database does not include hormone levels, pain scores, opioid consumption data, or biologic measurements necessary to evaluate mechanistic pathways directly.

No significant differences were observed in two-year reoperation rates, chronic knee pain, or postoperative complications such as SSI, deep infection, or arthrofibrosis. This may indicate that OCP-associated effects are more specific to postoperative recovery physiology rather than broad complication profiles or surgical failure rates. Taken together, these findings contribute to growing evidence suggesting that hormonal factors may influence ligament healing and pain sensitivity following ACLR.

Limitations

This study is limited by its retrospective design and reliance on an administrative database. OCP exposure was determined from prescription records and, therefore, could not confirm medication adherence, continuous use, formulation consistency, estrogen dose, or duration of therapy throughout the perioperative period. Additionally, patients using non-oral hormonal contraceptive methods may not have been fully identifiable, potentially introducing exposure misclassification. TriNetX does not record laterality, preventing confirmation of whether subsequent instability diagnoses or procedures were ipsilateral or contralateral. Furthermore, radiographic findings, physical examination data, patient-reported outcomes, and objective measures of knee laxity or graft maturation were unavailable.

Several clinically important confounders were not available within the database, including graft type, fixation technique, concomitant meniscal or cartilage procedures, rehabilitation adherence, surgeon variability, baseline activity level, and sport participation. Additionally, the broad age range included both adolescents and adults who may differ in skeletal maturity, hormonal physiology, injury mechanisms, and postoperative recovery patterns. However, this age range was selected to capture the population most commonly undergoing ACL reconstruction while maximizing cohort size and statistical power within a relatively uncommon exposure group. Differences in healthcare engagement between OCP users and non-users may also have influenced diagnosis capture and prescription reporting. Although propensity score matching controlled for measured confounders, unmeasured variables may still have influenced the observed associations.

Reoperation outcomes included heterogeneous knee procedures identified through CPT coding and could not be confirmed as directly related to graft failure or recurrent instability. Similarly, postoperative opioid use reflected database-recorded utilization and may have been influenced by prescribing practices, healthcare utilization patterns, pain severity, insurance factors, and healthcare access. Despite these limitations, the present study utilized a large multicenter propensity-matched cohort with standardized outcome definitions, allowing for the identification of clinically relevant associations that may help guide future prospective investigation into the relationship between hormonal exposure and postoperative ACLR outcomes.

## Conclusions

In this large propensity-matched cohort study, recorded perioperative OCP use was associated with lower rates of chronic knee instability diagnoses and fewer early postoperative opioid use following ACL reconstruction. However, causality cannot be established due to the retrospective observational design, and the biological mechanisms underlying these associations remain uncertain. These findings support further prospective investigation into the relationship between hormonal exposure and postoperative ACLR outcomes.

## Appendices

**Table 4:** CPT, ICD-10-CM, and RxNorm codes for TrinetX queries, inclusions, propensity score matching, and outcomes CPT: Current procedural terminology; ICD-10-CM: International Classification of Diseases, 10th Revision, Clinical Modification; BMI: Body mass index; SSI: Surgical site infection; ACL: Anterior cruciate ligament; MUA: Manipulation under anesthesia.

Item	CPT Code	ICD-10-CM Code	RxNorm
Arthroscopic ACL reconstruction (14-40 years old)	29888		
Ethinyl estradiol			4124
Estetrol			2539031
Norgestrel			7518
Norethindrone			7514
Desogestrel			22656
Drospirenone			11636
Levonorgestrel			6373
Norgestimate			31994
Ethynodiol			24591
Dienogest			22698
Mestranol			6782
*Exclusions*			
Unspecified multiple injuries		T07	
Osteoporosis with a current pathological fracture		M80	
Surgical procedures on the femur and the knee joint	1004987		
Pregnancy, childbirth, and the puerperium		O00-O9A	
Malignant neoplasms of bone and articular cartilage		C40-C41	
Arthroscopic knee procedures	1005641, 1005652, 1005657, 29870, 29888, 29889, M30-M36, N91, E28.2		
Systemic connective tissue disorders	M30-M36		
Amenorrhea/Oligomenorrhea	N91		
Polycystic ovarian syndrome	E28.2		
*Propensity score matching inputs*			
Osteoporosis without a current pathological fracture		M81	
Vitamin D deficiency		E55	
BMI > 30		Z68.3, Z68.4	
Tobacco use		Z72.0	
Diabetes mellitus		E08-E13	
Chronic kidney disease		N18	
Heart failure		I50	
*Outcomes*			
30-day SSI		T81.41	
Opioid use			5489, 6813, 7814, 7052, 3423, 7804, 6754, 480, 2670, 4337, 6813, 4166, 787390, 56795, 7242, 10689, 1551777, 29899, 6378, 7243, 7238, 1819, 3500, 6468, 73032
90-day deep/organ infections		T81.42-T81.44	
Six-month arthrofibrosis/MUA	29884, 27570		
Two-year reoperation	29884, 29877, 29874, 29880, 29881, 20680, 29876, 1005652, 1005657, 29888, 29889, 1005059, 27407		
Two-year chronic knee instability	M23.5		
Two-year chronic knee pain	M25.56		
